# Supplementary figures and images for: Phosphate starvation decouples cell differentiation from DNA replication control in the dimorphic bacterium Caulobacter crescentus
Source: PLoS Genet. 2023 Nov 27;19(11):e1010882. doi: 10.1371/journal.pgen.1010882 (PMC10723716; doi:10.1371/journal.pgen.1010882)

**A**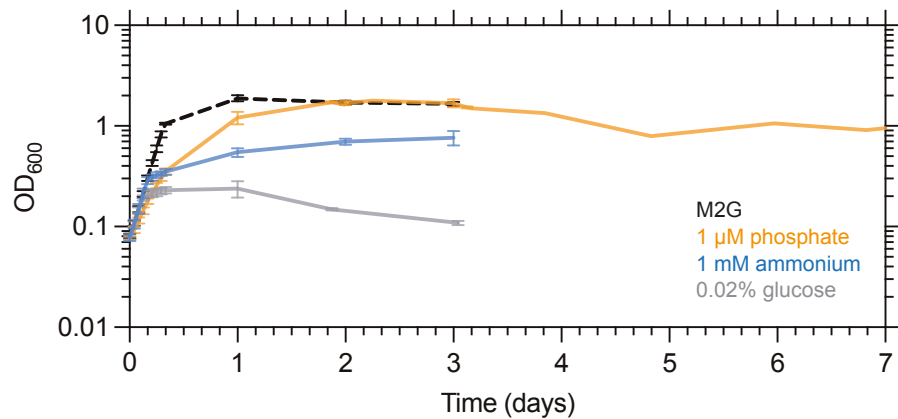**B**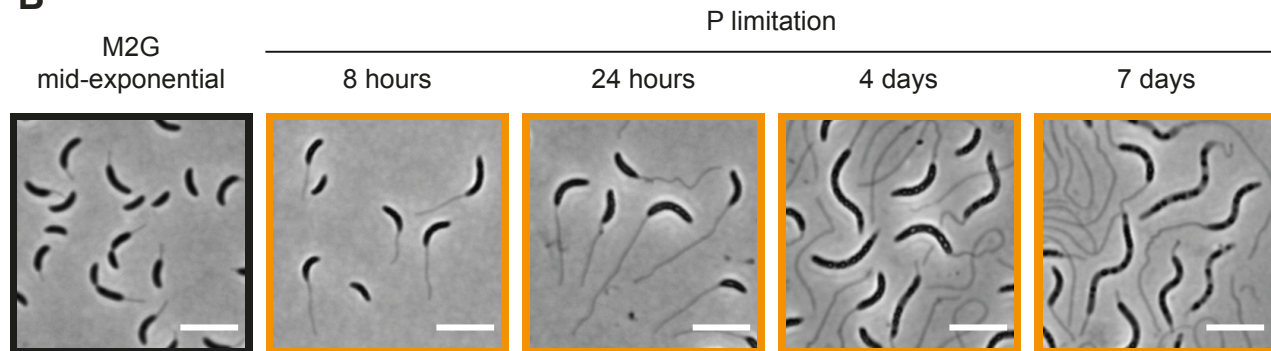

Supplement: S1 Fig — (A) Growth curves of wild-type cells in nutrient-replete M2G medium (black), and media limited for C (gray), N (blue), or P (orange). (B) Micrographs of cells in nutrient-replete M2G medium or after incubation in M5G 1 μM phosphate for the indicated amount of time. Scale bars: 5 μm. (PDF) [file pgen.1010882.s001.pdf]

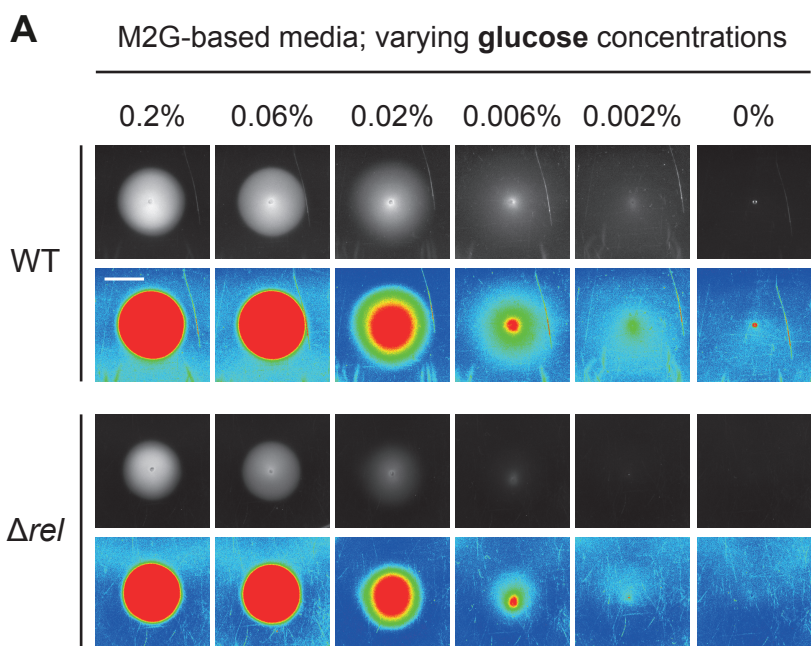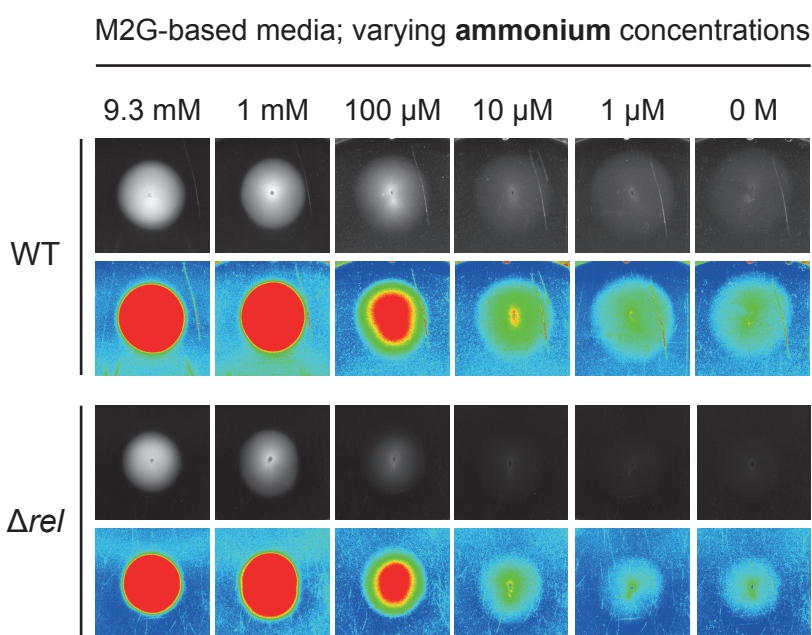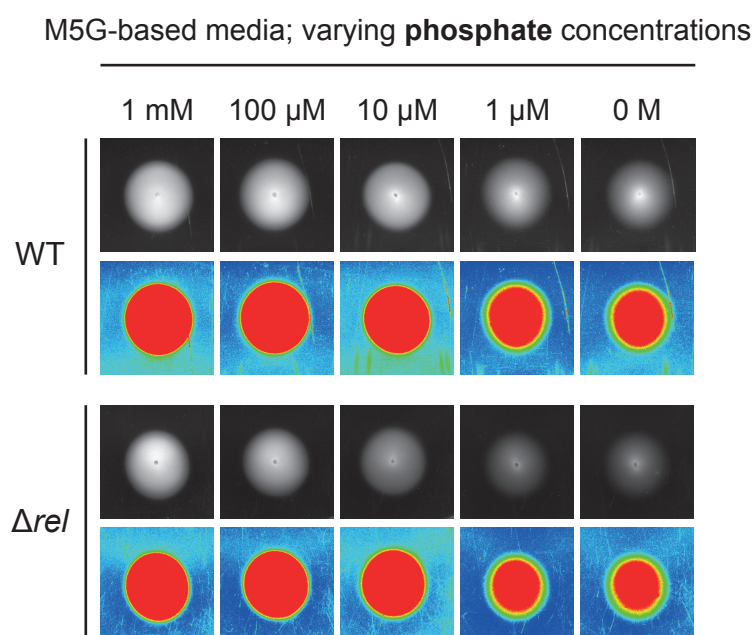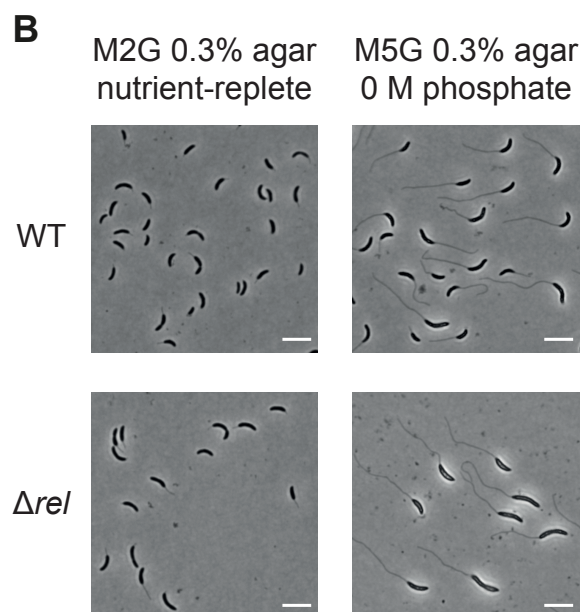

Supplement: S2 Fig — (A) Representative motility zone (soft agar colony) pictures in grayscale and the spectrum color lookup table of ImageJ (ver 2.1.0) after 72 hours incubation. All pictures are in scale (scale bar: 10 mm). (B) Micrographs of cells withdrawn from motility agar colony edges after 72 hours incubation, exhibiting clear phosphate starvation phenotypes in M5G prepared without phosphate. Scale bars: 5 μm. (PDF) [file pgen.1010882.s002.pdf]

**A**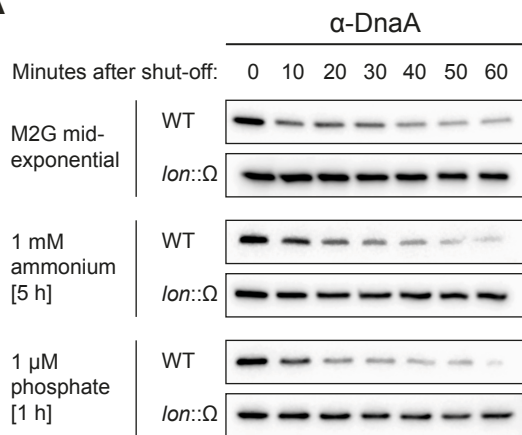**B**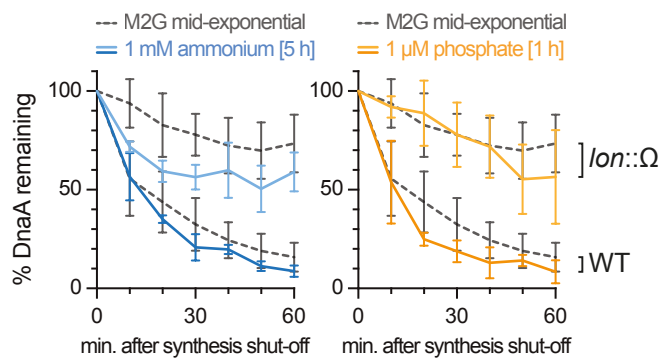**C**  $\alpha$ -DnaA, 0.02% glucose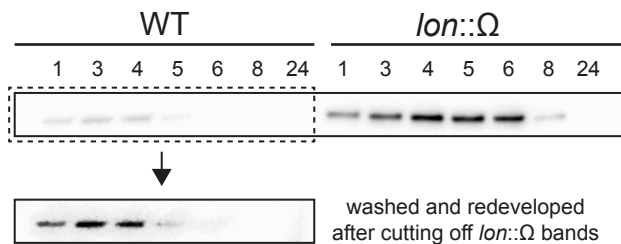**D**  $\alpha$ -DnaA, 1  $\mu$ M phosphate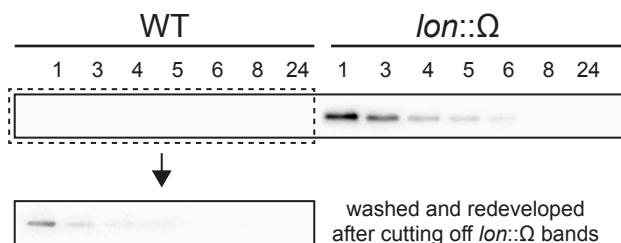**E**  $\alpha$ -DnaA, 1 mM ammonium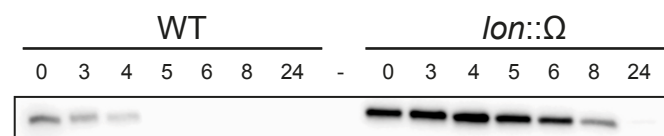**F**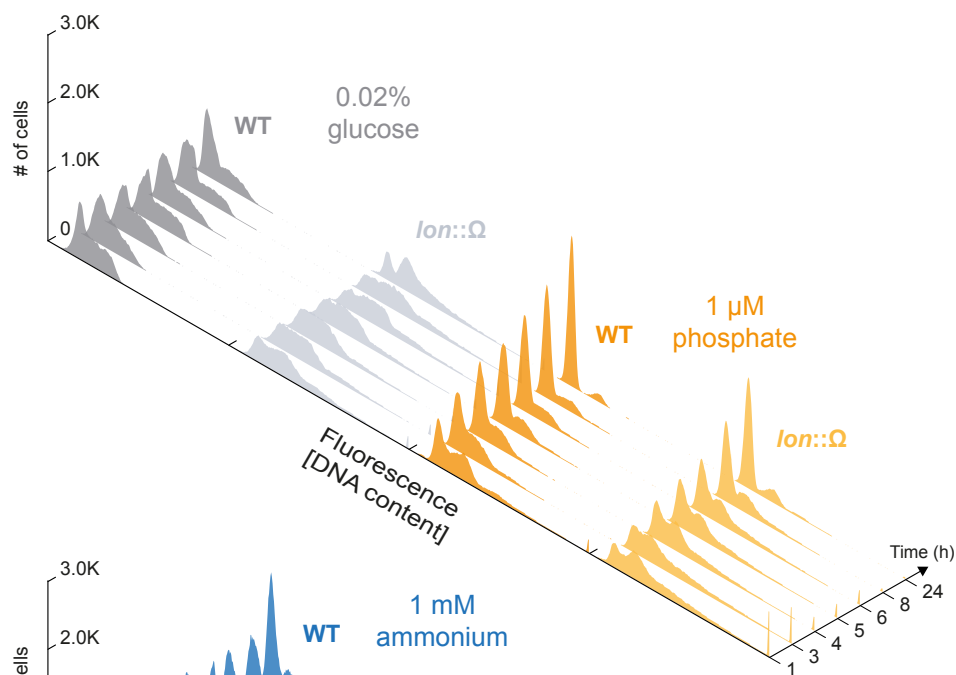**G**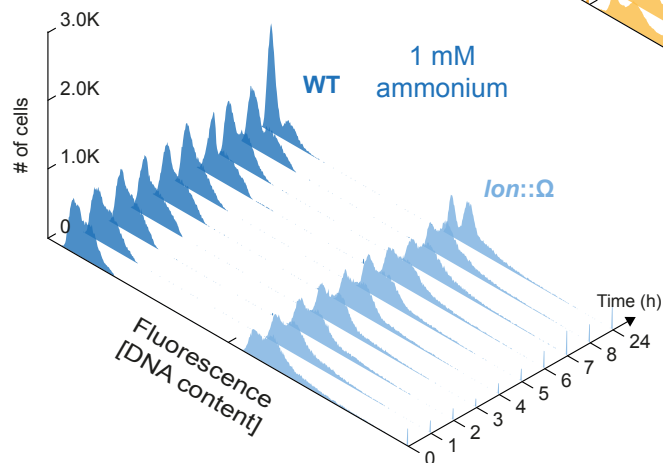

Supplement: S3 Fig — (A) DnaA in vivo stability assay of wild-type and lon::Ω cells in mid-exponential phase in M2G and while experiencing N or P starvation. (B) Quantification of DnaA in vivo stability assays done as shown in (A), from either five (M2G mid-exponential phase), two (N-limited), or three (P-limited) independent replicates for the lon::Ω strain, shown alongside wild-type data from Fig 3B, with error bars showing standard deviations. (C–E) Immunoblots of DnaA after shifting cells to nutrient-limited media, comparing lon::Ω to wild-type cells. For (C) and (D), the wild-type half of the blot was separated from the lon::Ω half, washed, and redeveloped to prevent the high signal of the lon::Ω bands from drowning out the wild-type signal. (F) DNA content as determined by flow cytometry of wild-type and lon::Ω cells after being shifted to C- or P-limited media. Each separate histogram represents 30 000 cells. (G) DNA content presented essentially as in (F), of wild-type and lon::Ω cells in mid-exponential phase in M2G before being shifted to N-limited medium (t = 0 h), and after being shifted to N-limited medium. (PDF) [file pgen.1010882.s003.pdf]

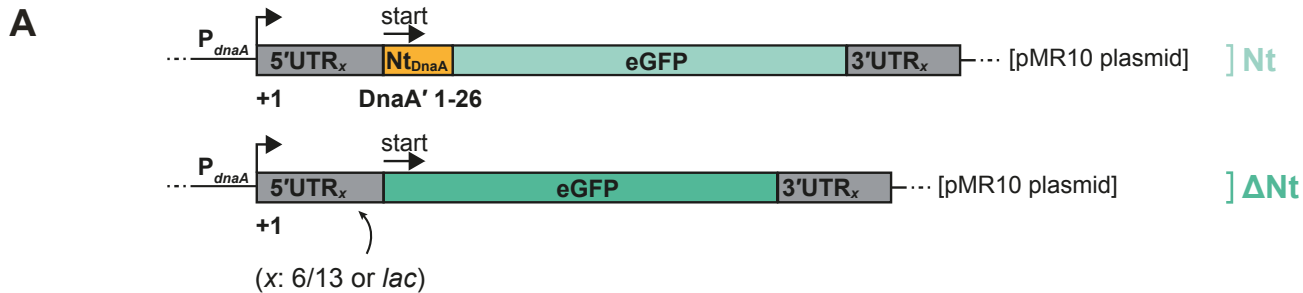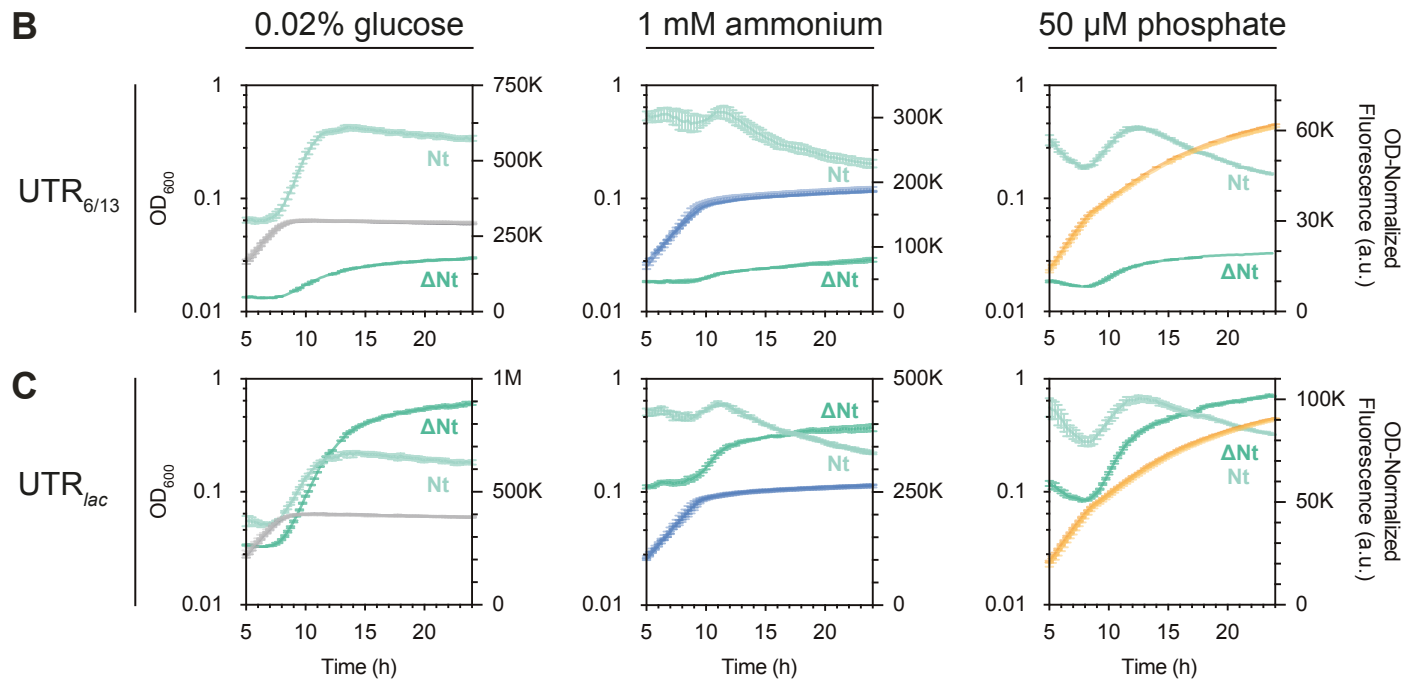

Supplement: S4 Fig — (A) Schematic depiction of dnaA expression reporter constructs from Felletti et al. [34]. (B–C) Growth curves (gray, blue, and orange) and OD600-normalized eGFP fluorescence (green) of cells harboring the dnaA expression reporter constructs depicted in (A), after shift to C-, N-, or P-limited media. Light hues: NtdnaA-eGFP constructs; dark hues: constructs with eGFP alone. eGFP-coding sequences are preceded by 5′ untranslated regions (5′UTRs) either of the artificial 6/13 UTR type (B), or from the lac operon of E. coli (C). (PDF) [file pgen.1010882.s004.pdf]

**A**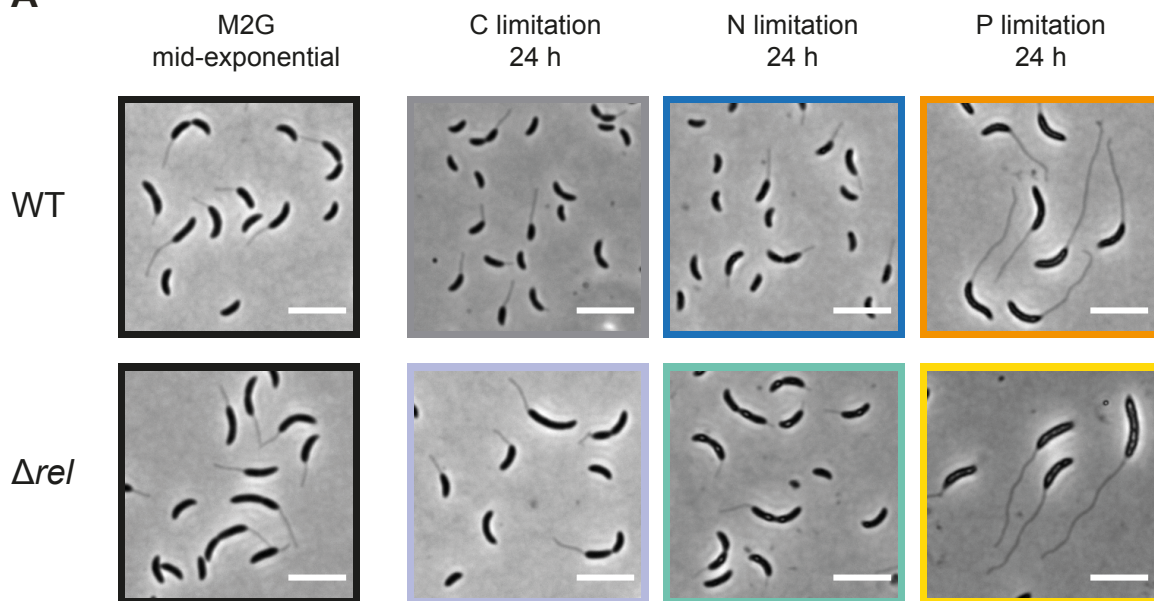**B**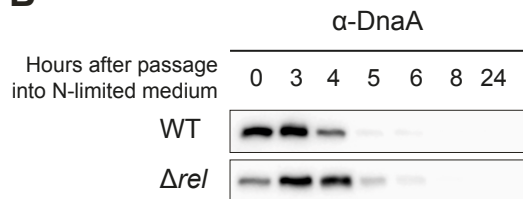**C**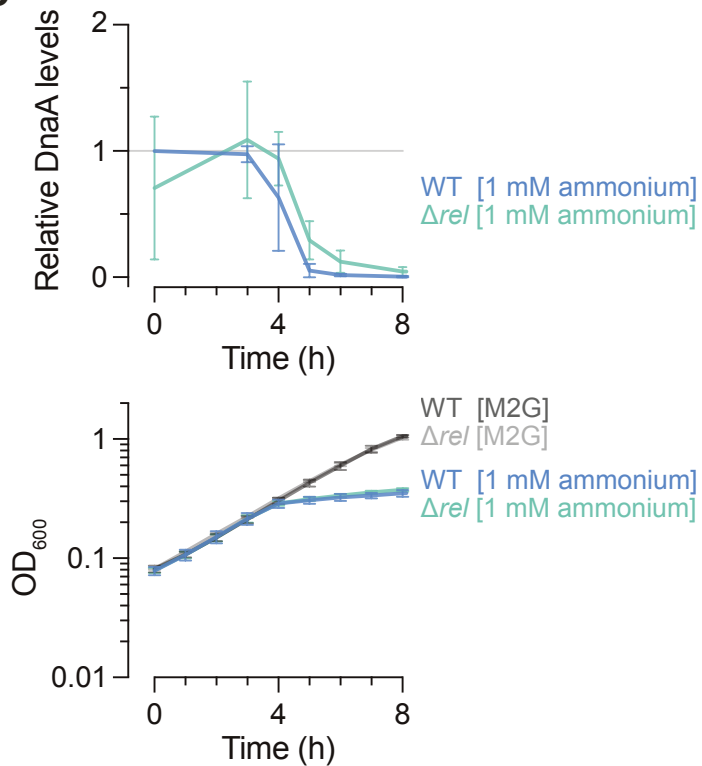

Supplement: S5 Fig — (A) Micrographs of Δrel cells sampled from a nutrient-replete culture exponentially growing at steady-state, and cells sampled after 24 hours in nutrient-limited media. Wild-type cells are shown for comparison. Scale bar: 5 μm. (B) Immunoblot of DnaA from nutrient-replete wild-type and Δrel M2G cultures in mid-exponential phase (t = 0 h) and after shifting cells to N-limited media. The blot picture has been cropped into two halves to juxtapose the two strains. (C) Quantification of DnaA levels in N-starved Δrel cultures (n = 3) compared to wild-type (n = 5). Mean DnaA levels are shown alongside growth curves of the Δrel strain in nutrient-replete minimal medium (n = 5), media limited for N (n = 10), and wild-type growth curves shown in Fig 1B. Error bars represent standard deviation. Δrel immunoblot samples were codeveloped with wild-type samples, and band intensities were normalized to t = 0 h bands of the wild type. (PDF) [file pgen.1010882.s005.pdf]

**A**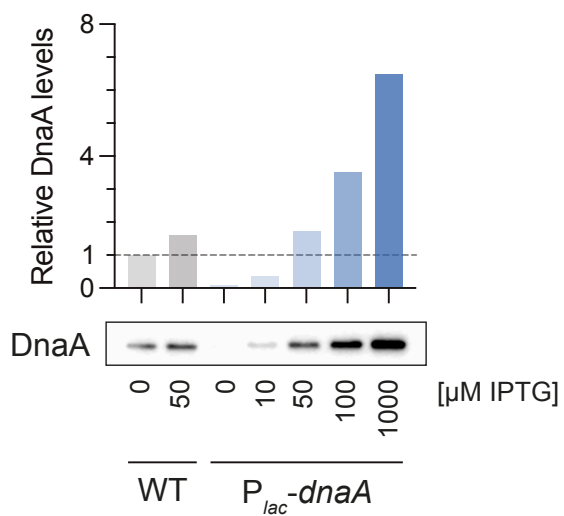**B**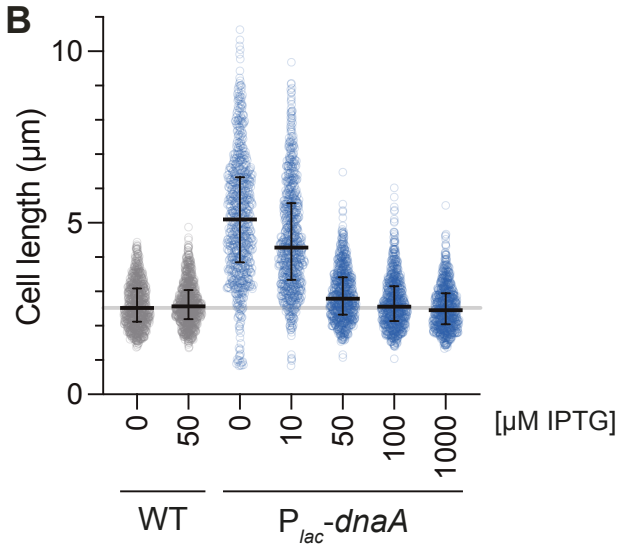**C**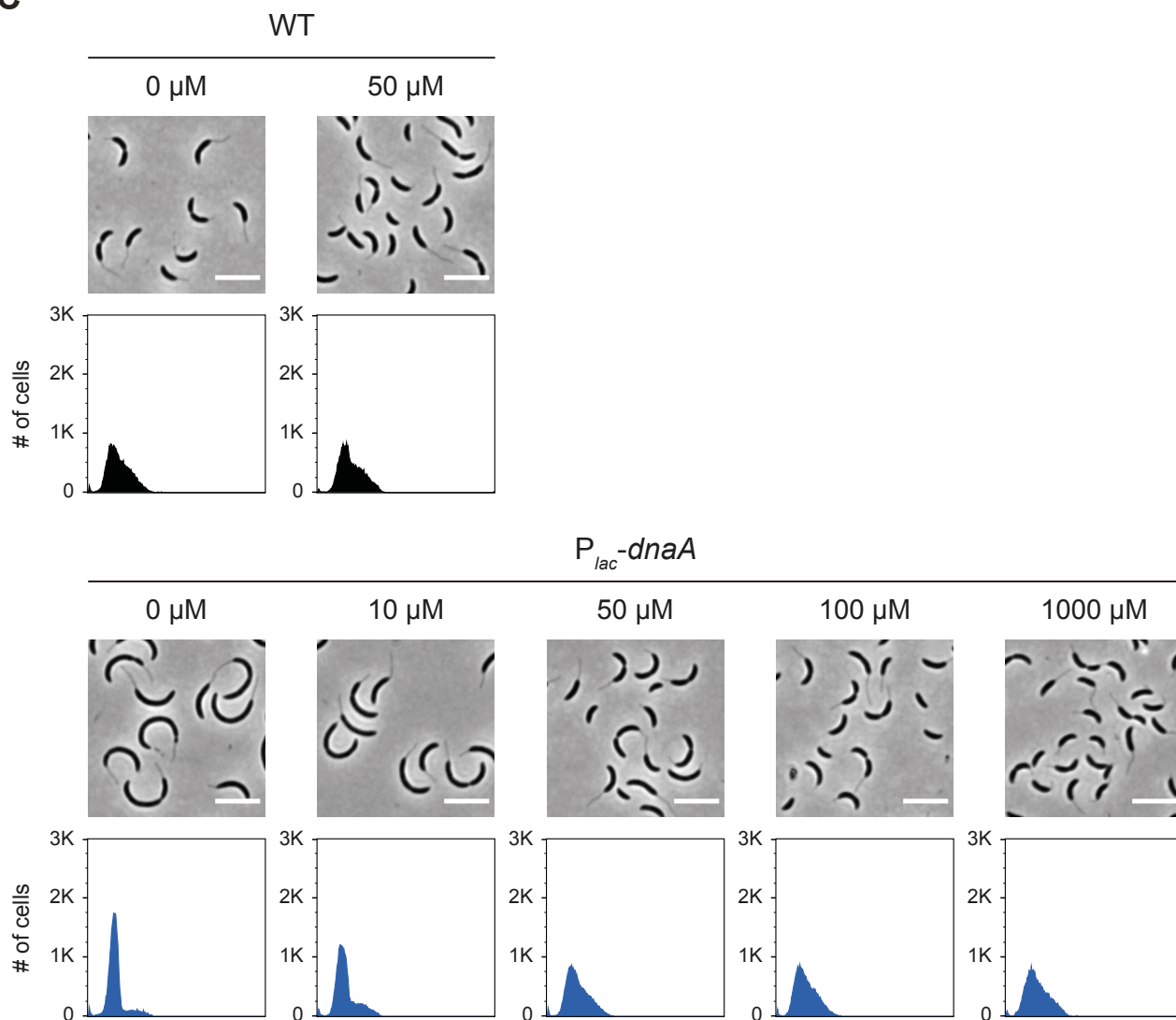

Supplement: S6 Fig — (A) DnaA immunoblot and respective quantification from wild-type and Plac-dnaA cells sampled at 4 hours after being shifted to M2G medium containing the indicated IPTG concentrations. Before shift, cells were cultivated until mid-exponential phase in M2G 50 μM IPTG. (B) Length measurements of 800 cells sampled as in (A). Lines and error bars indicate means ± SD. The extended horizontal line emphasizes the average length of wild-type cells in the absence of IPTG. (C) Micrographs and flow cytometry DNA content histograms (of 30 000 cells each) from cells sampled as in (A). Scale bars: 5 μm. (PDF) [file pgen.1010882.s006.pdf]

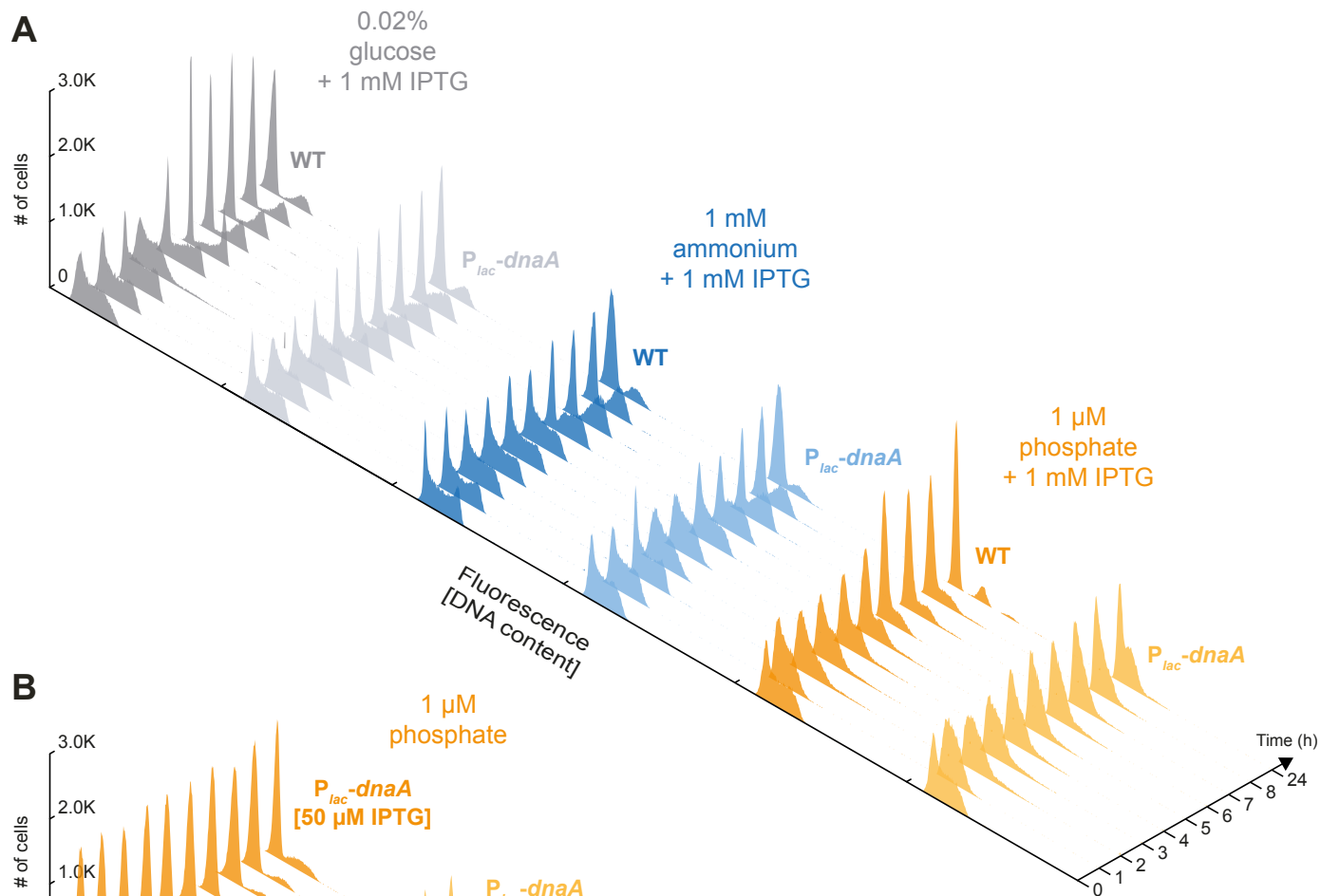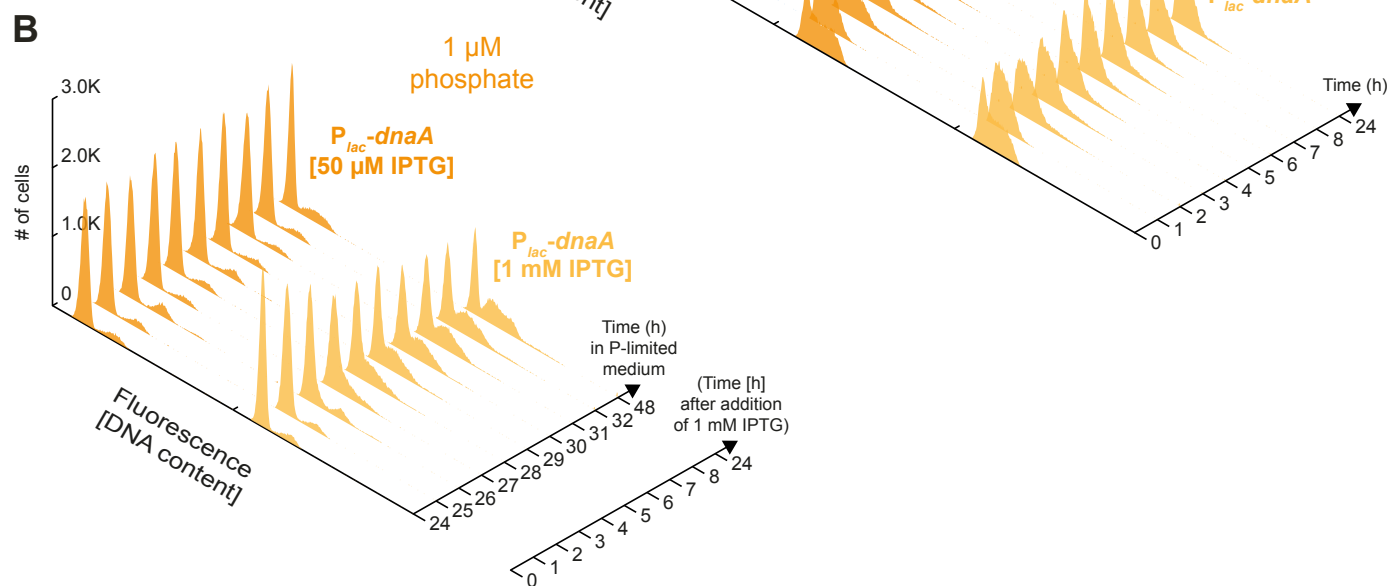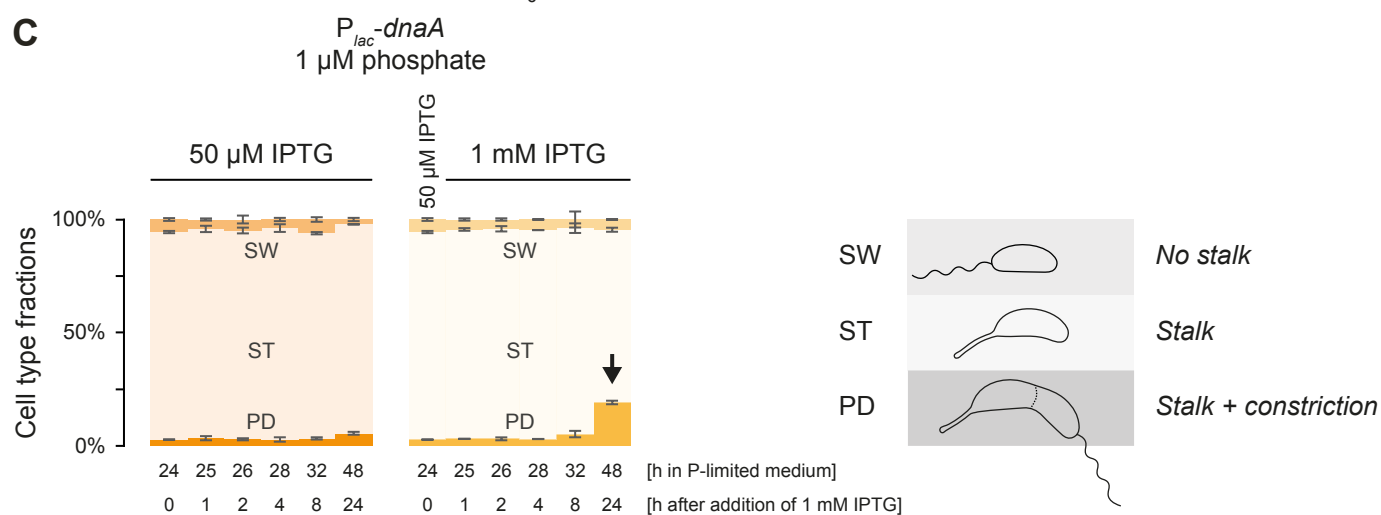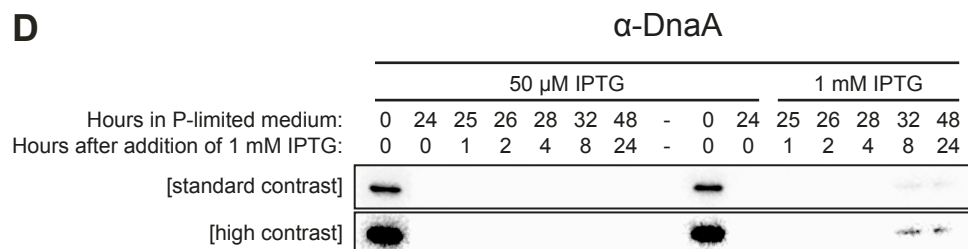

Supplement: S7 Fig — (A) DNA content as determined by flow cytometry of wild-type cells and Plac-dnaA cells in mid-exponential phase in M2G containing 50 μM IPTG (t = 0 h) and after being shifted to nutrient-limited media containing 1 mM IPTG to induce dnaA overexpression. P starvation data is also shown in Fig 5G. Each separate histogram represents 30 000 cells. (B) DNA content presented as in (A), of a Plac-dnaA culture being split after 24 hours in P-limited medium containing 50 μM IPTG (basal dnaA expression), with one culture half being supplemented with IPTG to a final concentration of 1 mM to boost dnaA expression. (C) Stacked bar chart showing the proportions of swarmer cells (SW), stalked cells (ST), and predivisional (PD) cells in Plac-dnaA cultures treated as in (B). Each bar represents the average of two biological replicates with error bars showing standard deviations. For each biological replicate, 586–1141 cells were counted (average: 801 cells). (D) Immunoblot showing DnaA levels of Plac-dnaA cells in mid-exponential phase in M2G containing 50 μM IPTG (0 h in P-limited medium) and after being treated as in (B). (PDF) [file pgen.1010882.s007.pdf]

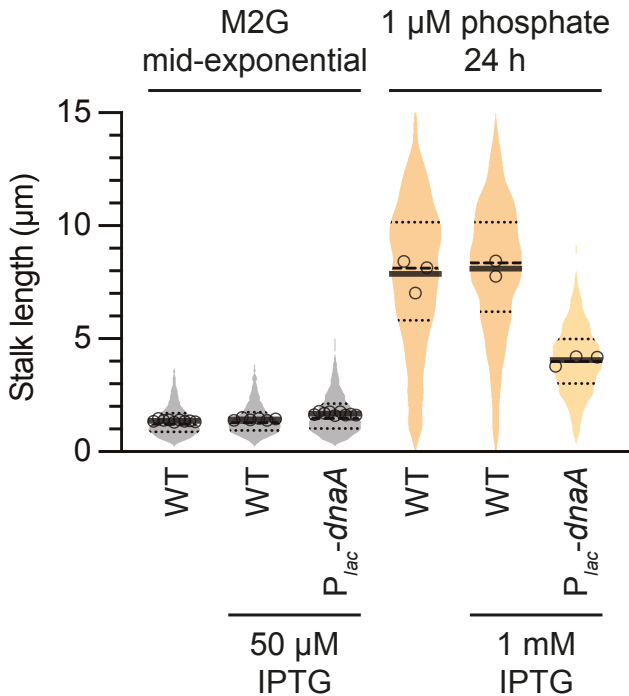

Supplement: S8 Fig — Stalk length violin plot of wild-type and Plac-dnaA cells in mid-exponential phase in M2G or after 24 h in P-limited medium in the presence of IPTG. Concentrations of the Plac inducer IPTG are indicated. Shown as done in Fig 1E, alongside stalk measurements of wild-type cells grown in absence of IPTG also shown in Fig 1E. Stalk measurements of cells grown in M2G containing 50 μM IPTG were done on six and nine biological replicates for wild-type and Plac-dnaA respectively, and for two and three biological replicates, respectively, for P-limited medium containing 1 mM IPTG. (PDF) [file pgen.1010882.s008.pdf]

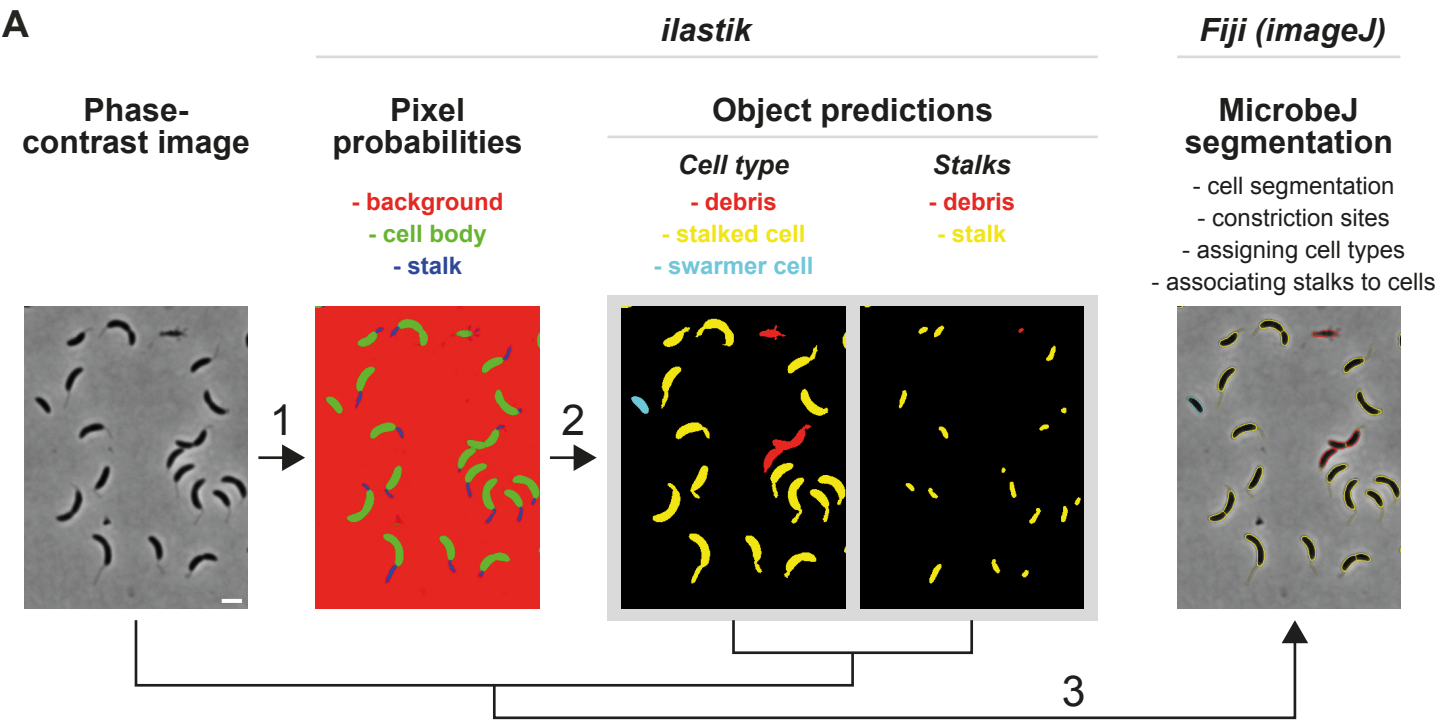

1

2

3

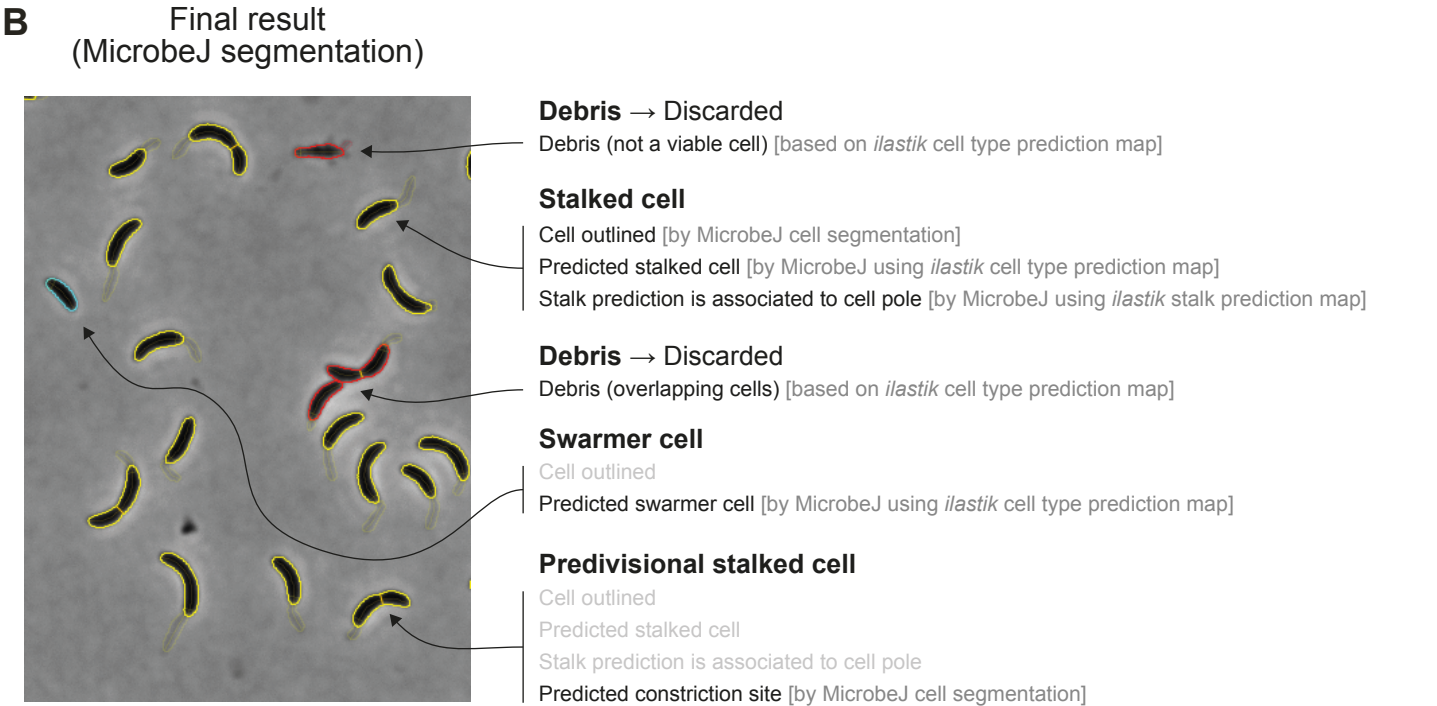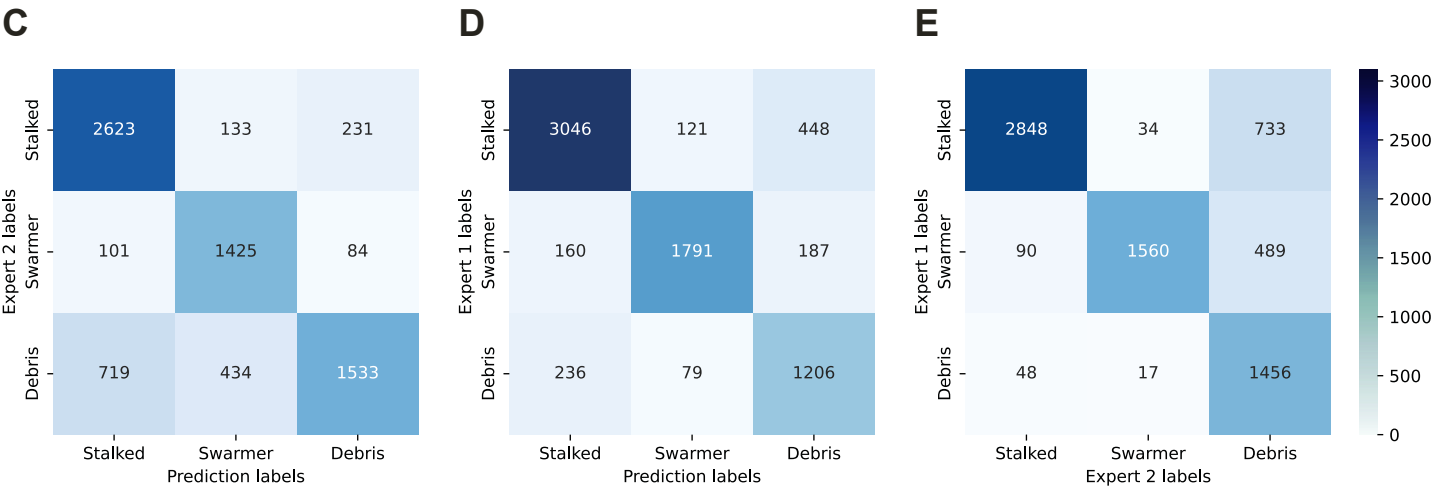

Supplement: S9 Fig — (A) Example images showing outputs from each major step of the machine learning-based automatic cell annotation of microscopy pictures. In step 1, pixels of phase-contrast images are classified as ‘background’, ‘cell body’, or ‘stalk’ in ilastik. In step 2, resulting pixel prediction maps are then used in ilastik to predict cell type objects and stalk objects, in two separate procedures. In step 3, cells are segmented and assigned constriction sites in MicrobeJ using the phase-contrast images. Segmented cells are then assigned cell types in MicrobeJ based on the cell type object prediction bitmaps generated by ilastik. Finally, stalks are assigned to the poles of segmented cells in MicrobeJ, using the stalk object prediction bitmaps generated by ilastik. Scale bar: 5 μm. (B) Zoomed-in view of the MicrobeJ output shown in (A), highlighting major annotated features. (C–E) Heat maps depicting confusion matrices comparing: (C) the manual annotations by Expert 1 to the predictions of the classification model, (D) the manual annotations by Expert 2 to the predictions of the classification model and (E) the manual annotations by Expert 1 to the manual annotations by Expert 2. Panels C, D, and E are plotted with the same color scale and contain a total of 7274, 7283 and 7275 observations, respectively. (PDF) [file pgen.1010882.s009.pdf]

$P_{dnaA}$ -5'UTR<sub>*dnaA*</sub>-Nt-eGFP-3'UTR<sub>*dnaA*</sub>

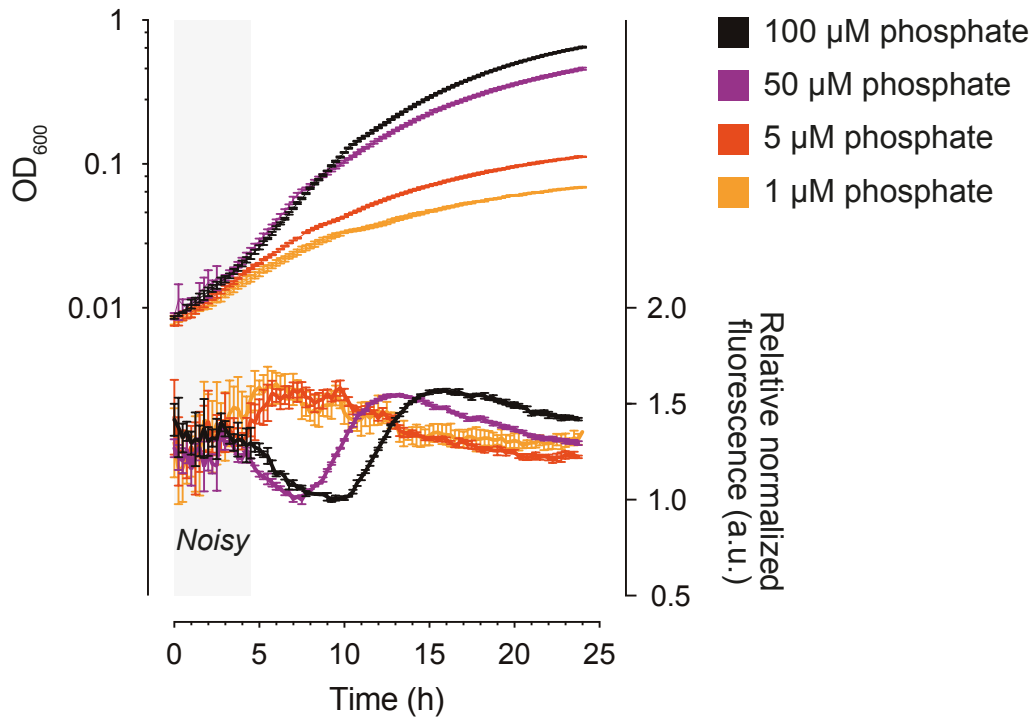

Supplement: S10 Fig — Triplicate growth curves and eGFP fluorescence measurements of the dnaA expression reporter construct “UTR-Nt” after shift to M5G with various initial phosphate concentrations. OD600-normalized eGFP fluorescence values were divided by the lowest observed OD600-normalized fluorescence measurement for each “phosphate concentration data set” (resulting in relative OD600-normalized fluorescence), to allow fluorescence patterns to be compared side-by-side. (PDF) [file pgen.1010882.s010.pdf]
