# Supplementary material for: Phosphate starvation decouples cell differentiation from DNA replication control in the dimorphic bacterium Caulobacter crescentus
Source: PLoS Genet. 2023 Nov 27;19(11):e1010882. doi: 10.1371/journal.pgen.1010882 (PMC10723716; doi:10.1371/journal.pgen.1010882)
Supplement: S1 Table — (PDF) [file pgen.1010882.s011.pdf]

Supplementary table

Table S1. Strains used in this study.

Strain name*	Genotype	Reference
NA1000	wild-type	(1)
KJ546 (KG329)	*lon*::Ω (spec^R)	Provided by Michael Laub, see (2)
KJ640 (ML2389)	Δ*rel* [Δ*spoT*]	(3)
KJ690 (ML2000)	*hfa*::P_{lacI}-*lacI* P_{dnaA}::P_{lac} [P_{lac}-*dnaA*]	(3)
BG	pMR10 [empty vector] (kan^R)	(4)
UTR	pMR10-P_{dnaA}-5′UTR_{dnaA}-*eGFP*-3′UTR_{dnaA} (kan^R)	(4)
UTR-Nt	pMR10-P_{dnaA}-5′UTR_{dnaA}-Nt_{DnaA}-*eGFP*-3′UTR_{dnaA} (kan^R)	(4)
6/13	pMR10-P_{dnaA}-5′$UTR_{6/13}$-*eGFP*-3′UTR_{dnaA} (kan^R)	(4)
6/13-Nt	pMR10-P_{dnaA}-5′$UTR_{6/13}$-Nt_{DnaA}-*eGFP*-3′UTR_{dnaA} (kan^R)	(4)
lac	pMR10-P_{dnaA}-5′UTR_{lac}-*eGFP*-3′UTR_{dnaA} (kan^R)	(4)
lac-Nt	pMR10-P_{dnaA}-5′UTR_{lac}-Nt_{DnaA}-*eGFP*-3′UTR_{dnaA} (kan^R)	(4)

*All strains are derivatives of the synchronizable holdfast-deficient *C. crescentus* strain NA1000 (CB15N).

Table S1 References

1. Evinger M, Agabian N. Envelope-associated nucleoid from *Caulobacter crescentus* stalked and swarmer cells. Journal of Bacteriology. 1977;132(1):294-301.

2. Omnus DJ, Fink MJ, Szwedo K, Jonas K. The Lon protease temporally restricts polar cell differentiation events during the Caulobacter cell cycle. eLife. 2021;10.

3. Leslie DJ, Heinen C, Schramm FD, Thüring M, Aakre CD, Murray SM, et al. Nutritional control of DNA replication initiation through the proteolysis and regulated translation of DnaA. PLOS Genetics. 2015;11(7):e1005342.

4. Felletti M, Romilly C, Wagner EGH, Jonas K. A nascent polypeptide sequence modulates DnaA translation elongation in response to nutrient availability. eLife. 2021;10:e71611.
