# Supplementary material for: Phosphate starvation decouples cell differentiation from DNA replication control in the dimorphic bacterium Caulobacter crescentus
Source: PLoS Genet. 2023 Nov 27;19(11):e1010882. doi: 10.1371/journal.pgen.1010882 (PMC10723716; doi:10.1371/journal.pgen.1010882)
Supplement: Attachment — Submitted filename: Point to point response_final.pdf [file pgen.1010882.s012.pdf]

Point-to-point response to reviewers (manuscript # PGENETICS-D-23-00834)

We thank the reviewers for the time they have invested to assess our work and provide these helpful and constructive suggestions. We have carefully considered their comments and have addressed each point either experimentally and/or by modifying the manuscript text to increase the clarity of our work. Please find our responses below, with the original points appearing in black and our replies in blue.

Comments to the Authors:

Reviewer #1: In this report by Hallgren, et al., the authors investigate the distinct responses to carbon, nitrogen, and phosphate limitation in Caulobacter crescentus. Cell cycle progression in Caulobacter involves both the regulation of DNA replication and the differentiation from stalked to swarmer cells. Here the authors demonstrate that limitation of any of these key nutrients results in translation-inhibition of the DNA replication regulator DnaA. Carbon and nitrogen starvation additionally lead to the production of the alarmone (p)ppGpp which inhibits the swarmer-to-stalk transition and leads to an accumulation of swarmer cells; by contrast, phosphate starvation does not increase (p)ppGpp and the cells overwhelmingly arrest in the stalked phenotype.

This work convincingly demonstrated that while C, N, and P starvation block DNA replication, cellular differentiation is regulated differently in C/N and P limited cells by (p)ppGpp. The increased motility of C/N starved Caulobacter cells is consistent with the phenotype observed in Vibrio and Pseudomonas species.

Overall, I found the experiments to be well designed and the conclusions followed the data. I do not have any serious concerns but offer the following suggestions that may enhance the manuscript.

1. In Figure 5B, it appears that IPTG-induced DnaA expression is not maintained over the time course for N starved cells. Was this observed consistently? Do you think this is due to overall effects on protein expression under N starvation, or is the induction weakening over time? I imagine the lac-promoter was chosen over xylose or vanillate since they would confound the C-starvation data. Could one of these promoters be used specifically for the N-limitation studies?

Response 1: We observed under all three starvation conditions, and in particular under N and P starvation, that after an initial IPTG-induced upregulation of DnaA, DnaA levels decreased again during prolonged starvation. This temporal pattern of DnaA accumulation was consistently observed and we explain it with the starvation-dependent translational regulation of *dnaA*, as described in Fig 3. The P_{lac}-*dnaA* strain still contains the Nt_{DnaA} sequence. Hence, we expect that the Nt_{DnaA}-dependent translational regulation of DnaA in combination with DnaA proteolysis counteracts P_{lac}-dependent transcription of *dnaA*. Re-doing the experiment with a different promoter is thus expected to lead to a similar temporal pattern of DnaA levels as obtained with the P_{lac} promoter.

Despite the transient nature of the DnaA accumulation, we think that our experimental setup is good enough to conclude that the general consequences of DnaA overproduction differ between P starvation and the other two starvation conditions. Under all three starvation conditions, we were able to boost DnaA synthesis enough to prevent DnaA from being cleared immediately following the onset of starvation (Fig 5B). In fact, DnaA levels remained relatively high under both C and N starvation even after 24 hours. This contrasts P starvation for which DnaA was completely cleared within 24 hours. The variation in DnaA accumulation between the three conditions in response to the same IPTG concentration is not unexpected, given the difference in growth kinetics (see Fig S1), and—as the reviewer alluded to—differences in global effects of starvation on transcription, translation, and energy metabolism. To enhance clarity, we have made changes to the Results section text that refers to Fig 5B.

2. In thinking about how the C/N and P limitation phenotypes are unique, I am curious if these phenotypes could be interconverted. For example, what would happen if (p)ppGpp levels were increased in P limited cells by overexpressing the constitutively active RelA allele from E. coli?

Response 2: We thank the reviewer for this relevant suggestion. We have tried to produce (p)ppGpp in P-starved cells by overexpressing RelA′-FLAG (alongside the "enzymatically dead" RelA′(E335Q)-FLAG

variant as a control) to see if this prevents differentiation from swarmer to stalked cells in P-starved cell. As shown below (**Revision Figure 2C**), most cells were still able to differentiate into stalked cells despite RelA′-FLAG overexpression. At first glance, this result could indicate that (p)ppGpp is not sufficient to cause swarmer cell accumulation under C and N starvation and that perhaps another factor is needed for blocking cell differentiation. However, at this point, we cannot rule out that the timing of RelA′-FLAG induction was inappropriate or that (p)ppGpp accumulated only to low levels or only transiently during the initial phase after induction of RelA′-FLAG expression. In fact, the relatively mild effect of RelA′-FLAG induction on growth (**Revision Figure 2A**), with doubling times mostly being affected during the initial 1–2 hours post-induction (**Revision Figure 2B**), could indicate that (p)ppGpp accumulation was not very efficient despite the high xylose concentration (0.3%) used. Consistently, we could only detect RelA′-FLAG by immunoblot with a weak and transient signal for the first and second hours post-induction (**Revision Figure 2D**). Since we currently do not have any assays set up in the lab to quantify (p)ppGpp accumulation, it is difficult for us to better control and further optimize this experiment. For this reason, we decided not to include these difficult-to-interpret RelA′-FLAG overexpression data in the manuscript. However, we have revisited the text passages in our manuscript to make sure that we do not imply that (p)ppGpp is sufficient for the swarmer cell accumulation under C/N starvation. Instead, we focus on our result that (p)ppGpp is required for the C and N-starvation induced swarmer cell accumulation, as shown in Fig 4.

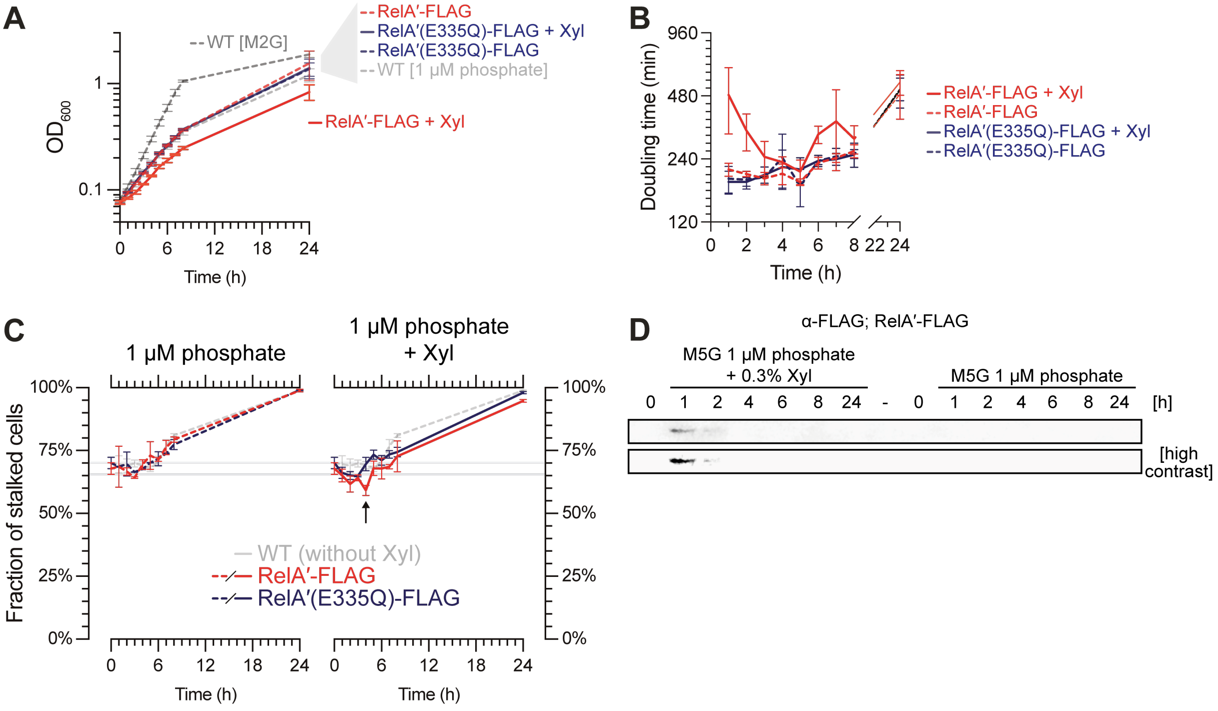

Revision Figure 2. Induction of RelA′-FLAG during P starvation. Constructs from Gonzalez and Collier (2014) expressing *relA′*-FLAG or the “enzymatically dead” *relA′*(E335Q)-FLAG allele from the *xylX* locus were subjected to P starvation (1 µM phosphate) in the absence or presence of 0.3% xylose (Xyl) to induce *relA′* construct expression. Growth was measured **(A–B)**, as well as the fraction of stalked cells **(C)** and RelA′-FLAG levels by immunoblotting **(D)**. The arrow in (C) highlights a potential transient accumulation of swamer cells (non-stalked cells). In all cases (except for the wild-type data borrowed from the manuscript), the average of three biological replicates, with error bars are shown.

3. For C/N limited cells, rel deletion increased the proportion of stalked cells. What would be expected if this mutation was combined with DnaA overexpression? Would the cells continue to divide until resources were completely exhausted? Would this lead to cell death rather that growth inhibition?

Response 3: This is another valuable suggestion. In order to assess the consequences of DnaA overexpression in the Δ*rel* mutant at the onset of carbon starvation, we have overexpressed DnaA from a plasmid-borne P_{lac}-*dnaA* construct in the wild-type and the Δ*rel* strain backgrounds. However, quantification of swarmer, stalked cell, and predivisional cell fractions using our ilastik image analysis workflow turned out to be challenging as some of the cells showed abnormal cell morphology (including filamentation), for which the ilastik workflow has not been trained, even under nutrient-replete uninduced

conditions (**Revision Figure 3A**). Hence, it is difficult to assess if cells continue to divide. Interestingly, however, when assessing the replication status of the Δ*rel* strain overexpressing *dnaA*, we observed a strong DNA replication over-initiation phenotype, which was absent in *dnaA*-overexpressing wild-type cells. This result is consistent with the notion that (p)ppGpp affects DNA replication initiation in a DnaA-independent manner (as indicated in our model Fig 6 and mentioned in the Discussion), for example by affecting CtrA or the supercoiling of the origin, as recently shown in *E. coli* (Kraemer et al., 2019, Fernández-Coll et al., 2020). While we think that our Δ*rel* P_{lac}-*dnaA* data are very interesting, we feel they may raise many new questions. Therefore, we have decided to not include them in this manuscript, and instead to work them out more thoroughly in a separate project.

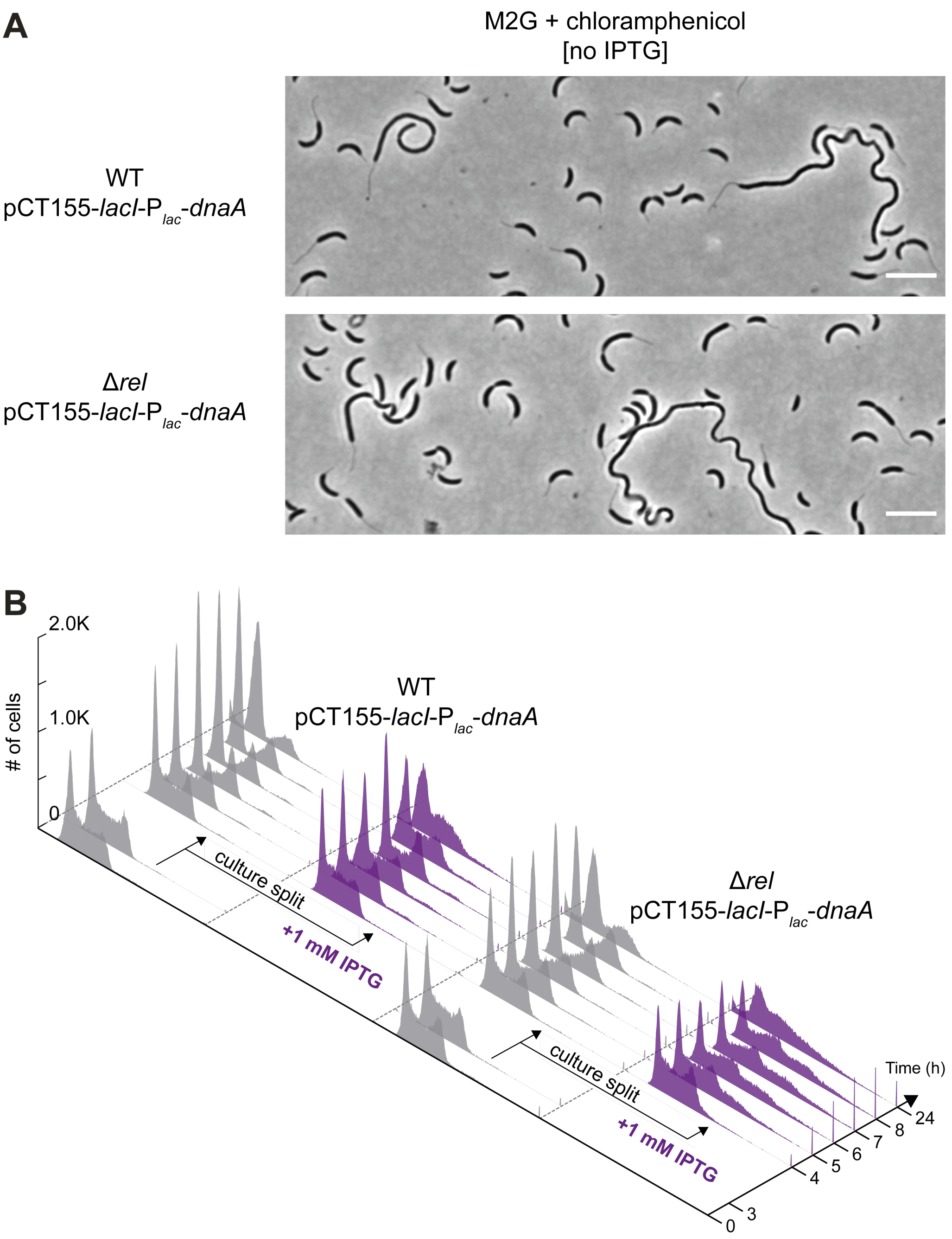

Revision Figure 3. (A) WT and Δ*rel* cells, harboring a plasmid-borne P_{lac}-*dnaA* construct, in mid-exponential growth in M2G medium. **(B)** One mid-exponential M2G culture of each of the two plasmid-bearing strains was subjected to C starvation for 3 hours before being split—with one culture half being induced with 1 mM IPTG. DNA content was measured by flow cytometry. All cultivation was done in media supplemented with 2 µg/ml chloramphenicol for plasmid maintenance.

4. Although these are distinct growth regimes, stationary phase entry shares features with nutrient limitation. The SpdS/SpdR two-component system is reported to be involved in stationary phase entry. Interestingly, these genes are upregulated upon carbon limitation (Britos et al. PLoS One, 2011). Since the signal required for activating SpdS is unknown, it may be interesting to assess whether this pathway is differentially regulated in the various nutrient limitations.

Response 4: This is indeed an interesting question, and we thank the reviewer for bringing the upregulation of SpdS/SpdR under stationary phase and carbon starvation to our attention. We agree that investigating the regulation of this two-component system under other forms of macronutrients will be very interesting. However, we believe that this question is not directly within the scope of this paper.

A few minor points:

1. As far as I am aware, in Caulobacter papers the enzyme required for (p)ppGpp production is referred to as SpoT. For consistency with existing literature, I would recommend replacing "Rel" with SpoT.

Response 5: As the reviewer points out, the (p)ppGpp hydrolase-synthase in *C. crescentus* has in several previous studies been referred to as SpoT. However, in this study we have decided to follow the phylogenetically consistent nomenclature for RSH family (RelA–SpoT homolog family) enzymes proposed by Atkinson et al. (2011) and Mittenhuber (2001); see also (Hauryliuk et al., 2015). In this nomenclature, the names "SpoT" and "RelA" are reserved for the two distinct enzymes present in most Beta- and Gammaproteobacteria, that are thought to have arisen through a gene duplication event from an ancestral "Rel" protein. Accordingly, for other bacteria, including Alphaproteobacteria like *C. crescentus*, the name Rel is used for RSH family enzymes. A previous study has already renamed *C. crescentus* SpoT to Rel (Shyp et al., 2020) and the Hallez lab has begun to use the more appropriate name Rel as well. To avoid confusion, we have added the previous name SpoT in parentheses behind the first mentioning of Rel in the text. To enhance clarity, we have now also added the former gene name to the figure legend of Fig 4 as well as the strain table (Table S1).

2. Line 277: The figure reference should be S5A.

Response 6: With this figure reference we are referring to all data shown in Fig S5; i.e., the Western blot data (Fig S5A), the cell length data (Fig S5B), and the flow cytometry and microscopy data (Fig S5C). Therefore, we changed the reference to Fig S5A–C (Fig S6A–C in the revised manuscript).

Reviewer #2: In this manuscript, Hallgren et al. characterise the physiological response of Caulobacter crescentus to carbon, nitrogen or phosphorus starvation. As expected, they showed that upon carbon (C) or nitrogen (N) limitation, both cell cycle progression and cellular differentiation are coordinately inhibited with swarmer cells remaining stuck in G1 and not differentiating into replication-competent stalked cells. In contrast, they found that phosphorus (P) depletion led to a cell cycle block similar to the one observed for C or N starvation but did not inhibit cell differentiation. Hence, C. crescentus starved for P accumulated stalked cells that are unable to initiate DNA replication. By determining the cellular levels of DnaA during nutrient exhaustion (C, N or P) and by comparing the WT to the ∆rel strain (unable to synthesise the second messenger (p)ppGpp), the authors concluded that cell cycle is likely controlled by a common mechanism regulating DnaA levels to nutrient availability while cell differentiation is rather under (p)ppGpp control.

Although many data are already separately available or strongly suggested in the literature, such as for example [(p)ppGpp accumulation during C or N but not P starvation, G1 block upon all the starvation conditions, cell differentiation and growth inhibition upon P limitation leading to elongated stalked cells, DNA levels dropping after nutrient depletion], this study has the advantage to bring all the data together facilitating the comparison between the stressful conditions. On the other hand, the difference observed between P and C or N starvation is very interesting, in particular knowing that Caulobacter likely often faces limitation for these macronutrients.

However, I would suggest to play a little bit more with (p)ppGpp and DnaA levels upon nutrient starvation to further support the two mechanisms behind the cell cycle/cell differentiation coordination.

1. Why did the authors evaluate the proportion of chromosome content by flow cytometry in the Plac-dnaA strain only starved for P and not for C or N? It might be interesting to check whether de novo

synthesis of dnaA can bypass the cell cycle block observed in C- or N-deplete conditions as it is the case for P starvation, even if the cell differentiation might still be blocked in a (p)ppGpp-dependent way.

Response 7: As suggested, we have included the flow cytometry data for these conditions as a new supplemental figure (Fig S7A). Consistent with our observations that the cell type proportions compared to the wild type are largely unaffected in the P_{lac}-*dnaA* strain limited for C or N in the presence of 1 mM IPTG, we do not see a notable change in cellular DNA content under these conditions. This indicates that DnaA clearance acts together with another mechanism to restrict DNA replication initiation under these conditions, as mentioned in the discussion.

2. Likewise, why didn't the authors test the ∆lon strain starved for N or P while they showed in a previous report this mutant could bypass the G1 block observed after carbon exhaustion. Measuring the chromosome content by flow cytometry in a ∆lon background after N or P exhaustion might be interesting as well.

Response 8: Based on the reviewer's suggestion, we have added new data for the *lon* mutant for N and P starvation. These data show that loss of Lon increases DnaA stability, consistent with the notion that Lon is the primary protease of DnaA. Similarly, we also observed that DnaA was maintained for a longer time during starvation in cells lacking Lon compared to the wild type. Under P starvation, the *lon* mutant was still able to efficiently clear DnaA, which we explain with continued growth under P starvation leading to a proportionally higher degree of DnaA dilution. These different kinetics in DnaA clearance are also reflected in the chromosome content of *lon* mutant cells after starvation for C, N, and P. While C- and N-starved cells still show a high number of cells with a chromosome content of > 1n, P-starved cells lacking Lon were still able to arrest with a single chromosome similar to the wild type. Altogether, these data are consistent with our conclusion that degradation of DnaA by Lon is required under all three starvation conditions to ensure fast DnaA clearance following the block of DnaA translation. The new data are presented in a new Fig. S3 of our manuscript and described in the section "DnaA is cleared via translation inhibition under diverse forms of starvation" of the Results.

3. It might also be interesting to test whether the inducible production of (p)ppGpp could inhibit the swarmer-to-stalked cell transition in P starved cells. This could be achieved either by using the tool developed by the Collier lab (pXTCYC-4-relA'-FLAG) or by concomitantly starving C. crescentus for P and N or C.

Response 9: As explained in our response to Reviewer #1 (Response 2), we have attempted to do the suggested (p)ppGpp overproduction experiment in P-starved cells. Due to difficulties to properly control this experiment, we did not include these data in the paper.

The alternative experiment, to concomitantly starve *Caulobacter* for P together with C or N, is unfortunately also very challenging in practice. The onset of starvation under our experimental conditions depends on the depletion of a particular nutrient through consumption of the nutrient by the active growth of the cells of the culture. To achieve this, we have used concentrations of glucose, ammonium, or phosphate that become growth-limiting when other nutrients are present at normal levels. Accordingly, if one macronutrient runs out, the consumption of the other nutrients will slow down or stop entirely. Thus, to ensure that a culture depletes two distinct macronutrients at the same exact time is unfortunately extremely difficult, or perhaps not even possible, as it would require a very precise nutrient composition and inoculation scheme.

4. Gorbatyuk et al (2005) showed that only N or C starvation led to a decrease of DnaA levels, not P limitation. This discrepancy should be at least discussed.

Response 10: Thanks for pointing that out. In the paper by Gorbatyuk and Marczynski (2005), the authors have shifted cultures from standard M2G medium to M2G completely lacking any P, thus removing the buffering system of the growth medium. This protocol is a very unconventional way of inducing P starvation and will likely affect other parameters of the growth medium, e.g., the pH. This paper also did not include additional controls to test if cells exhibit the typical characteristics of P-starved cells (long stalks, elongated cell bodies). It is also worth to mention that our result is consistent with a paper by the Thanbichler lab (Billini et al., 2019), which showed that DnaA and CtrA are cleared after prolonged P starvation. Although we think that the data presented by Gorbatyuk and Marczynski do not

challenge our results and conclusions, we agree that the discrepancy between the data should be discussed and we have added a sentence in the manuscript to do this.

5. Britos et al (2011) showed that carbon starvation uncouples swarmer cell differentiation and the G1-to-S phase transition. They mentioned that upon carbon, the flagellum was ejected and the stalk biogenesis initiated but not completed making the stalk invisible by light microscopy. Hence, swarmer cells starved for carbon were erroneously considered as swarmer cells while in fact these were differentiated stalked cells with the stalks visible by electron microscopy. Since the cell type were determined based on light microscopy images and that cells were considered as swarmer cells because they were stalkless, the authors should check the presence of the flagellum by electron microscopy. And if flagella are detected, the discrepancy between both studies should be discussed.

Response 11: This finding by Britos et al. (2011) is indeed interesting. However, we think that it is a consequence of the experimental condition that had been chosen. In contrast to the nutrient exhaustion experiments, that we and others have conducted to assess starvation responses under physiologically relevant conditions, Britos et al. shifted cultures from M2G medium to M2, completely lacking any carbon. Our own data indicate that this condition results in the complete block of cellular activities after a short delay, i.e., cells are unable to complete DNA replication and cell division, and just arrest the cell cycle at the stage where they are (**Revision Figure 4**). In the Britos et al. experiment, the authors shifted synchronized swarmer cells to M2. These cells had likely just enough resources to initiate the differentiation into stalked cells before they were locked in this state due to the lack of energy. Hence, a population of very early stalked cells is expected to occur. Whether these cells have initiated DNA replication is hard to assess with the ParB reporter (shown in Figure 4 of Britos et al. (2011)), as the replicated origins might not have been translocated yet. For all these reasons, we think that the phenomenon described by Britos et al. (2011) is a consequence of shifting swarmer cells to M2 medium and thus does not apply to our physiologically more relevant nutrient exhaustion experiments. Our data showing a swarmer cell accumulation under C starvation are consistent with data published by Boutte and Crosson that showed that the proportion of cells without a polar fluorescent focus of DivJ-YFP increases upon C starvation (Boutte et al., 2012), a stalked cell-associated histidine kinase that localizes at the old cell pole during cell differentiation. Furthermore, the consistency between our motility and microscopy data further supports an accumulation of swarmer cells. That being said, visualizing the flagellum would be a very valuable additional control for our cell type quantifications. Unfortunately, we do not have the infrastructure and experimental setup available to do this additional test for this revision. However, as the editor suggested, we have added a paragraph in the manuscript (discussion, end of first section) to discuss limitations of our image analysis workflow.

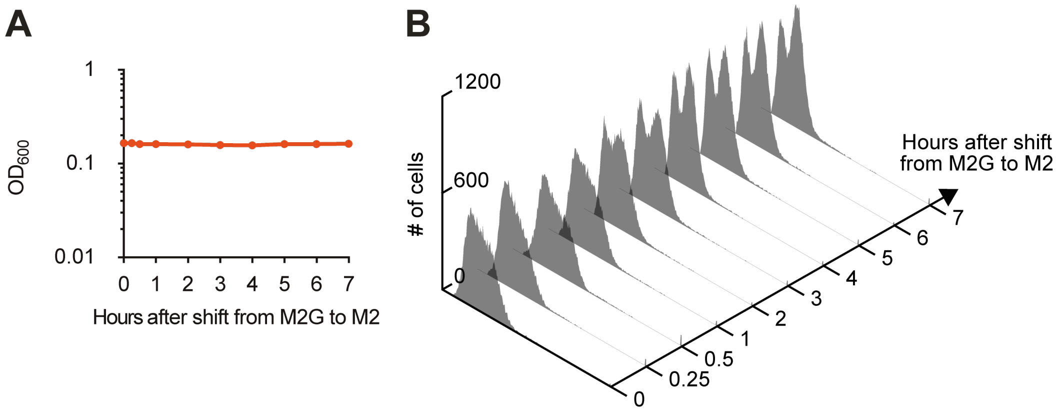

Revision Figure 4. Growth **(A)** and cellular DNA content as determined by flow cytometry **(B)** after shifting a mixed population of cells from mid-exponential phase in M2G to M2 completely lacking glucose.

Minor comments

- p4 (L69): the authors wrote “However, they are arrested in the G1 phase and must differentiate into a stalked cell in order to enter S phase and to complete the cell cycle”. In fact, cell differentiation is not a prerequisite for cell cycle progression, so the swarmer cells do not have to differentiate into stalked cells

to enter S phase. There are mutants unable to eject flagellum and/or to synthesise a stalk which can still start DNA replication initiation and ultimately divide.

Please rephrase to remove the mandatory sense of the sentence.

Response 12: Thanks for pointing this out. We have rephrased this sentence.

- p12 (L234): the word "weakened" should be replaced by "decreased" or "diminished".

Response 13: As the reviewer suggested, we have replaced "weakened" by "decreased".

Reviewer #3: Overall, the work is well executed and clearly presented. It is bizarre that DnaA is cleared so much faster under P limitation compared to C and N limitation without change in stability. The authors showed that for a GFP translational reporter, the 5'UTR is required for downregulation under P starvation, but they have not shown that this region is sufficient and would still confer control under P limitation when grafted downstream of a promoter, other than PdnaA, for example Plac. In fact, Plac expression overrides the P starvation-induced reduction of DnaA to a certain extent, therefore it is evident that promoter control via PdnaA also contributes to DnaA levels and this could be specifically addressed. So far, it is only demonstrated that the UTR confers P-starvation control in the context of the native PdnaA promoter.

Response 14: Our data show that the region downstream of the *dnaA* start codon referred to as Nt_{DnaA} (not the 5′UTR!) is needed for DnaA downregulation at the onset of the three different starvation conditions. The involvement of this Nt_{DnaA} region to the starvation-dependent regulation of DnaA abundance was first described in our previous work (Felletti et al., 2021). This study showed that this Nt_{DnaA} sequence is not only required but also sufficient (regardless of promoter or 5′UTR) to reduce translation from a DnaA translational reporter, even in the heterologous host *E. coli* (Fig. 6A–B in Felletti et al. (2021). However, this previous study was exclusively focused on the role of this sequence under C starvation. Our new data demonstrate that the same sequence is also involved in the downregulation of DnaA at the onset of N and P starvation.

Additionally, we show that DnaA is degraded by the Lon protease under all three starvation conditions (see new Fig S3 and **Response 8**), but that the rate of proteolysis is not increased under starvation. As the reviewer correctly states, expression of *dnaA* from P_{lac} can partially override the starvation-dependent clearance of DnaA. Importantly, this does not mean that the promoter of DnaA is nutritionally controlled. The net cellular DnaA levels are the product of the rate of synthesis and proteolysis. We think that strongly boosting *dnaA* transcription counteracts the Nt_{DnaA}-dependent reduction of DnaA translation efficiency, allowing DnaA to be synthesized fast enough to prevent its otherwise rapid clearance by basal proteolysis. However, the strong Nt_{DnaA}-dependent inhibition of translation in combination with proteolysis are expected to outpace transcription over time, which is indeed reflected in the reduction of DnaA levels over time despite strong *dnaA* transcription (Fig. 5B).

Based on the findings described in Fig. 3, we think it is appropriate to conclude that DnaA is cleared by a similar mechanism (i.e., inhibited DnaA translation in combination with continued proteolysis) under the different starvation conditions. It may appear strange that DnaA clearance occurs much faster under P starvation than under N and C starvation. However, we think that these differences in how fast DnaA is cleared are due to the different growth kinetics under the different conditions. While N and C starvation result in a complete growth arrest, P-starved cells are still able to accumulate biomass. Thus, under P starvation, growth-dependent dilution further contributes to the clearance of DnaA. We have added a discussion of this aspect of DnaA dilution under P starvation in the new text describing the newly added *lon* mutant data (see **Response 8**).

I would also like to know if DNA replication can be rescued in P-starved bearing Plac-dnaA, in which IPTG induction occurs after 24h of starvation when the culture only contains 1N cells.

Response 15: As the reviewer suggested, we have tested if inducing P_{lac}-*dnaA* expression after 24 hours of P starvation can still induce DNA replication and have added the resulting data to our manuscript, Fig. S7B–D. These data show that IPTG addition at this late time point during P starvation resulted only in mild DnaA accumulation. Nevertheless, this mild increase of DnaA levels was sufficient

to trigger DNA replication initiation, and subsequently cell division, in a fraction of cells. This result provides additional support of our conclusion that DnaA is limiting for DNA replication initiation and cell cycle progression during P starvation.

Line 364 “Our new work revealed that DnaA regulation occurs by the same mechanism under N and P starvation.” - And yet the abundance of DnaA during the time course seems substantially different …. ??

Response 16: As explained in **Response 14** as well as **Response 1**, we explain the starvation-dependent differences in the kinetics of DnaA clearance with the different growth behaviors and metabolic statuses under the three starvation conditions. Since P-starved cells still continue to substantially accumulate biomass, the block of DnaA synthesis in combination with proteolysis result in faster DnaA clearance than in growth-arrested C and N starved cells. That being said, we agree with the reviewer that our choice of words can be improved. To better reflect the differences between the starvation conditions, we have revised the text passages that mention the commonality of the mechanisms of DnaA clearance under various starvation conditions, and replaced the phrase “same mechanism” by “similar mechanism”.

References

ATKINSON, G. C., TENSON, T. & HAURYLIUK, V. 2011. The RelA/SpoT Homolog (RSH) Superfamily: Distribution and Functional Evolution of ppGpp Synthetases and Hydrolases across the Tree of Life. *PLoS ONE,* 6**,** e23479.

BILLINI, M., BIBOY, J., KÜHN, J., VOLLMER, W. & THANBICHLER, M. 2019. A specialized MreB-dependent cell wall biosynthetic complex mediates the formation of stalk-specific peptidoglycan in *Caulobacter crescentus*. *PLOS Genetics,* 15**,** e1007897.

BOUTTE, C. C., HENRY, J. T. & CROSSON, S. 2012. ppGpp and polyphosphate modulate cell cycle progression in *Caulobacter crescentus*. *Journal of Bacteriology,* 194**,** 28-35.

BRITOS, L., ABELIUK, E., TAVERNER, T., LIPTON, M., MCADAMS, H. & SHAPIRO, L. 2011. Regulatory Response to Carbon Starvation in Caulobacter crescentus. *PLoS ONE,* 6**,** e18179.

FELLETTI, M., ROMILLY, C., WAGNER, E. G. H. & JONAS, K. 2021. A nascent polypeptide sequence modulates DnaA translation elongation in response to nutrient availability. *eLife,* 10**,** e71611.

FERNÁNDEZ-COLL, L., MACIAG-DORSZYNSKA, M., TAILOR, K., VADIA, S., LEVIN, P. A., SZALEWSKA-PALASZ, A. & CASHEL, M. 2020. The Absence of (p)ppGpp Renders Initiation of Escherichia coli Chromosomal DNA Synthesis Independent of Growth Rates. *mBio,* 11**,** 10.1128/mbio.03223-19.

GONZALEZ, D. & COLLIER, J. 2014. Effects of (p)ppGpp on the progression of the cell cycle of *Caulobacter crescentus*. *Journal of Bacteriology,* 196**,** 2514-2525.

HAURYLIUK, V., ATKINSON, G. C., MURAKAMI, K. S., TENSON, T. & GERDES, K. 2015. Recent functional insights into the role of (p)ppGpp in bacterial physiology. *Nature Reviews Microbiology,* 13**,** 298-309.

KRAEMER, J. A., SANDERLIN, A. G. & LAUB, M. T. 2019. The Stringent Response Inhibits DNA Replication Initiation in E. coli by Modulating Supercoiling of <i>oriC</i>. *mBio,* 10**,** 10.1128/mbio.01330-19.

MITTENHUBER, G. 2001. Comparative genomics and evolution of genes encoding bacterial (p)ppGpp synthetases/hydrolases (the Rel, RelA and SpoT proteins). *J Mol Microbiol Biotechnol,* 3**,** 585-600.

SHYP, V., DUBEY, B. N., BÖHM, R., HARTL, J., NESPER, J., VORHOLT, J. A., HILLER, S., SCHIRMER, T. & JENAL, U. 2020. Reciprocal growth control by competitive binding of nucleotide second messengers to a metabolic switch in *Caulobacter crescentus*. *Nature Microbiology,* 6**,** 59-72.
